# Malaria Epidemiology and *Plasmodium* Species–Specific Antimalarial Treatment Patterns Among RDT–Confirmed Cases in Northwestern Pakistan

**DOI:** 10.3390/tropicalmed11070194

**Published:** 2026-07-10

**Authors:** Aqsa Mansoor, Ghulam Narjis, Imen Ben Abdelmalek, Sabika Firasat, Iffat Naz, Kiran Afshan

**Affiliations:** 1 Department of Zoology, Faculty of Biological Sciences, Quaid-i-Azam University, Islamabad 45320, Pakistan; 2 Department of Statistics, Rawalpindi Women University, 6th Road, Satellite Town, Rawalpindi 46300, Punjab, Pakistan; 3 Department of Biology, College of Science, Qassim University, Buraydah 51452, Saudi Arabia

**Keywords:** malaria, epidemiology, drug effect, public health, Pakistan

## Abstract

Background: Malaria remains a major public health challenge in Pakistan, where persistent transmission, heterogeneous risk patterns, and evolving treatment practices continue to impede control and elimination efforts. Methods: A community-based cross-sectional study was conducted among 9211 symptomatic individuals in District Dera Ismail Khan, northwestern Pakistan, from January 2024 to December 2025. Malaria was diagnosed using rapid immunochromatographic assays for *Plasmodium vivax* (*P. vivax*) and *Plasmodium falciparum* (*P. falciparum*), and associated risk factors were assessed using multivariable logistic regression. Results: Overall malaria prevalence was 36.27% (3341/9211). *P. vivax* predominated, accounting for 93.8% (3133/3341) of infections, while *P. falciparum* represented 5.8% (195/3341) and mixed infections 0.4% (13/3341). Infection risk was significantly associated based on multivariate analysis with male sex, younger age, pregnancy (AOR = 4.69), and absence of mosquito net use (AOR = 6.17), whereas indoor residual spraying (AOR = 0.08) showed a strong protective effect. Malaria transmission showed two peaks: October–December (AOR = 3.78–4.97) and February–March (AOR = 2.25–2.31) and declined significantly based on unadjusted analysis from 39.5% in 2024 to 32.8% in 2025 (OR = 0.74; *p* < 0.001). Blood groups B+ (AOR = 0.77), B− (AOR = 0.78), and AB− (AOR = 0.86) were significantly associated with reduced odds of malaria infection. A notable shift toward artemisinin-based combination therapy was observed, with artemether–lumefantrine largely replacing chloroquine for malaria treatment. Conclusions: The persistence of *P. vivax*-dominated transmission alongside ongoing *P. falciparum* circulation highlights the need for targeted vector control, enhanced surveillance, and evidence-based treatment strategies to accelerate malaria elimination in Pakistan.

## 1. Introduction

*Plasmodium* is a eukaryotic protozoan parasite responsible for malaria, a vector-borne disease transmitted through the bite of an infected female *Anopheles* mosquito [1,2]. Although approximately 250 *Plasmodium* species have been identified, 5 species commonly infect humans: *P. falciparum*, *P. vivax*, *Plasmodium ovale*, *Plasmodium malariae* and *Plasmodium knowlesi* (a zoonotic species). Among these, *P. falciparum* and *P. vivax* are the most prevalent [3]. Malaria occurs year-round, although its incidence varies seasonally [4]. It remains a major global health burden and is ranked among the leading causes of death worldwide. According to the WHO World Malaria Report 2025, an estimated 282 million malaria cases and approximately 610,000 deaths were reported globally in 2024 across 80 malaria-endemic countries and territories, representing a 3% increase in cases compared with 2023 [5]. The majority of cases (around 88%) occur in the African region, while approximately 12% are reported from the Eastern Mediterranean region. In the Eastern Mediterranean Region, six high-burden countries account for most of the malaria transmission, with Sudan contributing 44.6% of the estimated burden, followed by Pakistan, Yemen, Somalia, Afghanistan, and Djibouti. The estimated increase in malaria burden is multifactorial, where complex emergencies, population displacement, and climate-related crises continue to disrupt health systems and malaria control interventions [6].

Malaria remains a major public health challenge in Pakistan, with an estimated 1.8 million cases reported in 2025, a substantial population residing in endemic areas [7]. Although *P. vivax* accounts for most infections, *P. falciparum*, responsible for more severe disease manifestations, remains an important public health concern [8,9]. Transmission dynamics in Pakistan vary markedly across regions, largely influenced by local climatic and ecological conditions [10]. Environmental disruptions, particularly the catastrophic floods of 2022, have further intensified malaria transmission. Heavy monsoon rains from June to August severely impacted Khyber Pakhtunkhwa study province, creating ideal breeding conditions for vectors and overwhelming public health infrastructure [11,12,13].

Accurate and timely diagnosis is fundamental to effective malaria management, enabling prompt treatment, limiting disease progression, and preventing unnecessary antimalarial use in parasite-negative individuals [14]. Rapid diagnostic tests (RDTs) have significantly advanced malaria detection in resource-limited settings where microscopy is often unavailable or impractical [15]. These immunochromatographic assays detect species-specific Plasmodium antigens directly from whole blood, providing reliable results within 15–30 min without requiring specialized equipment or highly trained personnel [16]. At the point of care, RDTs such as the *P.f./P.v.* Antigen Rapid Test Cassette allow accurate species differentiation by targeting *P. falciparum* histidine-rich protein-2 (HRP-2) and *P. vivax* lactate dehydrogenase (Pv-LDH) [17].

Despite diagnostic advances, effective treatment remains indispensable for preventing progression to severe and life-threatening malaria [18]. The World Health Organization (WHO)recommends artemisinin-based combination therapies (ACTs) as first-line treatment for uncomplicated *P. falciparum* malaria, while chloroquine continues to be used for *P. vivax* in many endemic regions. ACTs combine a fast-acting artemisinin derivative with a longer-acting partner drug to ensure complete parasite clearance and minimize recrudescence, with commonly used regimens including artemether–lumefantrine, artesunate–amodiaquine, and dihydroartemisinin–piperaquine [19]. Primaquine is an essential component of *P. vivax* malaria management because it provides radical cures by eliminating dormant hepatic hypnozoites and reducing the risk of relapse when used alongside blood-stage antimalarial therapy [2]. However, the emergence and spread of resistance to chloroquine, sulfadoxine–pyrimethamine, and more recently artemisinin threatens global malaria control efforts [20]. Continuous therapeutic efficacy monitoring is therefore critical to detect early resistance and guide policy updates; WHO recommends revising first-line therapies when treatment failure rates exceed 10%, ensuring sustained treatment effectiveness and improved patient outcomes [5].

Understanding local epidemiological patterns and treatment practices is essential for improving malaria control strategies and reducing disease burden. However, limited information is available regarding the epidemiology of malaria and species-specific antimalarial treatment patterns in this region. Therefore, the present study aimed to investigate malaria epidemiology and assess species-specific antimalarial treatment patterns among rapid diagnostic test (RDT)-confirmed malaria cases in northwestern Pakistan.

## 2. Materials and Methods

### 2.1. Study Area

**Figure 1:**
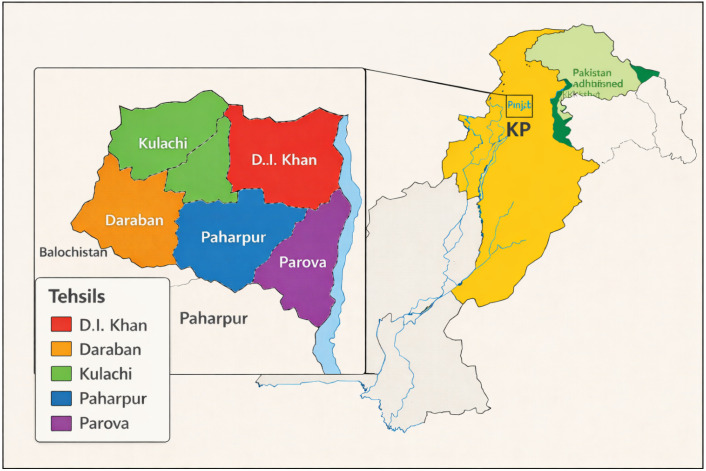
Map of Pakistan showing the sampling district of Dera Ismail Khan and its administrative subdivision (Kulachi, D.I.K., Daraban, Paharpur, and Parova).

Dera Ismail Khan is a district located in the southern region of Khyber Pakhtunkhwa province, Pakistan (Figure 1). The region is characterized by a semi-arid climate and is considered vulnerable to vector-borne diseases due to poor drainage. A community-based cross-sectional study was carried out at the (DHQ) District Headquarter Hospital Dera Ismail Khan. All five administrative subdivisions and surrounding boundaries of district D.I.K.: Kulachi, D.I.K., Daraban, Paharpur, and Parova were chosen for the current study to record the prevalence of malaria. The study sites included the 114 Medical centers, Laboratories, Clinics and Hospitals under DHQ Dera Ismail Khan.

### 2.2. Study Design, Participants Recruitment and Sample Size

A community-based cross-sectional study was conducted from January 2024 to December 2025 in District Dera Ismail Khan, Khyber Pakhtunkhwa, Pakistan.

Participants were recruited from selected healthcare facilities and community screening sites representing different geographical areas of the district. Individuals presenting with clinical features suggestive of malaria were screened, and eligible participants were enrolled after obtaining informed consent. Epidemiological and clinical information was collected using a structured questionnaire (Supplementary File S1) followed by malaria diagnosis using rapid diagnostic testing (RDT). The required sample size was calculated using the formula described by Daniel [21] *n* = *Z^2^P*(1 − *P*)/*d*^2^, where n represents the required sample size, *Z* represents the confidence level, *P* represents the expected prevalence, and *d* represents the desired precision. A 95% confidence level (*Z* = 1.96), an expected prevalence of 50% (*P* = 0.50), and a precision level of 1% (*d* = 0.01) were used for sample size estimation. The prevalence value of 50% was selected to obtain the maximum sample size because a precise local prevalence estimate was not available before the study.

The study included 9211 participants from District Dera Ismail Khan, subdivision Kulachi (*n* = 5382), D.I.K. (*n* = 2630), Daraban (n = 619), Paharpur (*n* = 380), Parova (*n* = 21), and Adjacent boundaries (*n* = 179). The number of participants recruited from each site was determined according to population coverage, malaria transmission intensity, accessibility, healthcare utilization patterns, and availability of suspected malaria cases during the study period.

### 2.3. Ethical Approval and Informed Consent

The study protocol was reviewed and approved by the Institutional Ethical Review Committee of Quaid-i-Azam University, Islamabad) and the district headquarter officer (DHO) of DHQ Hospital Dera Ismail Khan (Approval No.1909). The study was conducted in accordance with ethical principles for research involving human participants. Written informed consent was obtained from all adult participants before enrollment. For participants below the age of consent, informed consent was obtained from parents or legal guardians.

### 2.4. Questionnaire and Participants Inclusion Criteria

A total of 9211 patients of all ages and both sexes presenting with malaria-like symptoms (fever, headache, chills, nausea, fatigue, vomiting, abdominal pain, myalgia, joint pain, and diarrhea) were enrolled in the study. Participants were included if they were presented with malaria-compatible symptoms, agreed to participate, and provided informed consent. Participants with incomplete clinical information, unavailable diagnostic results, or those refusing participation were excluded from the study. Patient-level data were collected using a structured questionnaire administered alongside rapid diagnostic testing (RDT). The questionnaire comprised five sections covering sociodemographic characteristics (age, sex, education, and occupation), clinical symptoms, preventive practices, environmental risk factors, and antimalarial drug prescribed/used.

Collected epidemiological and clinical data were reviewed for completeness and consistency before analysis. Data was entered into a standardized database and checked for errors. Records with missing key clinical information, diagnostic results, or incomplete questionnaire responses were excluded from the respective analyses. Only complete records were included in all analyses.

### 2.5. Blood Collection and Rapid Immunochromatographic Assay

Whole blood samples were obtained by finger-prick or venipuncture (EDTA tubes). Samples were analyzed immediately after collection or stored at 2–8 °C for up to 72 h prior to testing. Malaria infection was diagnosed using a commercially available immunochromatographic rapid diagnostic test (RDT) (HEALGEN, Catalog No. GCMAL (pf/pv)-402a; Healgen Scientific LLC, Houston, TX, USA), Malaria *P. falciparum/P. vivax* Antigen Rapid Test Cassette (Whole Blood), which qualitatively detects histidine-rich protein 2 (HRP-2) specific to *P. falciparum* and parasite lactate dehydrogenase (Pv-LDH) specific to *P. vivax*. The assay utilizes monoclonal antibodies immobilized on a nitrocellulose membrane to capture parasite antigens, with a built-in control line to confirm proper test performance. For each test, 5 µL of whole blood was added to the sample well followed by three drops (~120 µL) of assay buffer, and results were interpreted after 20–30 min according to the manufacturer’s instructions.

**Figure 2:**
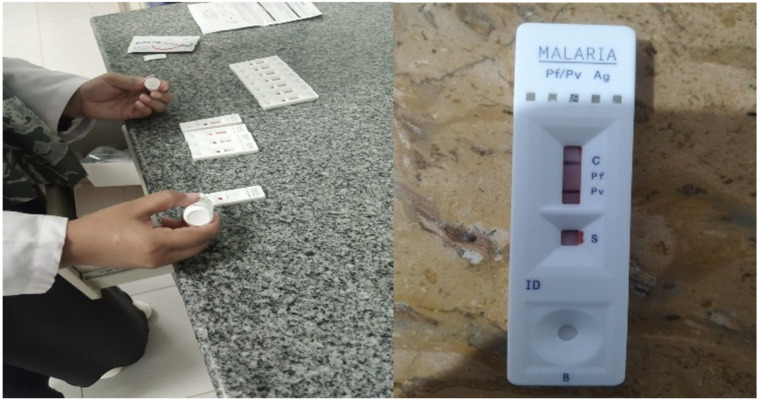
Immunochromatographic malaria rapid diagnostic test (RDT) cassette used for qualitative detection of *P. falciparum* (HRP-2) and *P. vivax* (Pv-LDH) antigens in whole blood samples.

Although microscopy of Giemsa-stained blood smears is considered the reference method for malaria diagnosis, RDTs were used in this study due to their rapid turnaround time, minimal infrastructure requirements, and suitability for resource-limited healthcare settings. The selected *Pf/Pv* RDT was appropriate because *P. falciparum* and *P. vivax* are the dominant malaria species circulating in Pakistan and represent the primary species requiring differentiated treatment approaches. The selected assay enables rapid differentiation between *P. falciparum* and *P. vivax* under field conditions, making it suitable for large-scale epidemiological studies in resource-limited settings. However, detection of less common species including *P. malariae*, *P. ovale*, and *P. knowlesi* was not assessed in this study, as their identification requires molecular or advanced diagnostic approaches. All tests were performed following standard laboratory procedures, and assay validity was verified by the internal control line (Figure 2). As the test provides qualitative detection only, parasite density could not be determined, and results were interpreted in conjunction with clinical findings.

### 2.6. Statistical Analysis

Data was entered and managed in Microsoft Excel, and statistical analyses were conducted using R (version 4.5.2) and SPSS (version 20). Descriptive statistics were used to summarize the demographic and clinical characteristics of the study population. Categorical variables were entered into logistic regression models with epidemiologically meaningful reference categories. Odds ratios (ORs) with 95% confidence intervals (CIs) were calculated for comparisons relative to the reference category (baseline). Reference categories were not assigned ORs because they represent the baseline level (OR = 1). Variables demonstrating significant associations (*p* ≤ 0.05) in the univariate analysis and biologically relevant from existing literature were subsequently included in a multivariable logistic regression model to identify independent predictors while controlling potential confounding effects. Adjusted odds ratios (AORs) with 95% CIs were calculated to estimate the strength of associations. Multicollinearity was checked using Variance Inflation Factor (<5), and statistical significance was defined at *p*-value ≤ 0.05.

## 3. Results

In the current study, malaria afflicted 36.27% of the patients with malaria-like symptoms in 5 subdivisions of D.I.K. and surrounding areas (3341/9211), with *P. vivax* accounting for 34% (3133/9211). A total of 2.1% (195/9211) patients had *P. falciparum*, and 13 had mixed infections.

### 3.1. Malaria and Sociodemographic Features

Table 1 represents the participants’ sociodemographic and environmental risk factors associated with malaria infection. Female sex was significantly associated with reduced odds of malaria infection compared with males (OR = 0.84; 95% CI 0.77–0.91; *p* = 0.0001). Compared with individuals aged 1–20 years, older age groups had progressively lower odds of malaria infection, with significant reductions observed in those aged 21–40 years (OR = 0.66; 95% CI 0.61–0.73; *p* < 0.001) and 41–60 years (OR = 0.60; 95% CI 0.50–0.73; *p* < 0.001), while the association for those aged >60 years was not statistically significant (OR = 0.63; 95% CI 0.37–1.07; *p* = 0.089). Linked with adjacent areas, residents of Dera Ismail Khan (OR = 1.61; 95% CI 1.13–2.30; *p* = 0.0079), Daraban (OR = 2.37; 95% CI 1.62–3.48; *p* < 0.001), Kulachi (OR = 1.94; 95% CI 1.36–2.75; *p* = 0.0002), and Paharpur (OR = 2.15; 95% CI 1.43–3.21; *p* = 0.0002) had significantly higher odds of malaria, while the association for Parova was not statistically significant (OR = 1.63; 95% CI 0.61–4.30; *p* = 0.3234). Using children as the reference group, the odds of malaria infection were significantly lower among farmers (OR = 0.65; 95% CI 0.54–0.78; *p* < 0.001), housewives (OR = 0.62; 95% CI 0.50–0.70; *p* < 0.001), labour (OR = 0.79; 95% CI 0.68–0.91; *p* = 0.0015), and those in other occupations (OR = 0.77; 95% CI 0.60–0.99; *p* = 0.0441). Individuals with a history of travel had significantly higher odds of malaria infection (OR = 1.81; 95% CI 1.12–2.94; *p* = 0.0148), indicating that recent travel was associated with an increased risk of malaria.

The proportion of cases was slightly higher during the rainy season (37.9%) compared with the dry season (36.0%); however, this difference was not statistically significant (OR = 1.08, 95% CI: 0.93–1.24; *p* = 0.2846). Similarly, bed net use showed no significant association, with comparable proportions observed among users (36.8%) and non-users (36.0%) (OR = 1.03, 95% CI: 0.92–1.14; *p* = 0.5656). In contrast, the presence of a water reservoir was strongly associated with increased risk, as households with water reservoirs exhibited a higher proportion of cases (37.0%) than those without (33.5%), corresponding to more than threefold increased odds (OR = 3.33, 95% CI: 3.00–3.70; *p* < 0.001). Annual spraying showed a significant protective effect, with a substantially lower proportion of cases among sprayed households (21.0%) compared with those without spraying (39.5%), representing a 58% reduction in odds (OR = 0.42, 95% CI: 0.37–0.47; *p* < 0.001).

### 3.2. Temporal Factors Linked to Malaria

Malaria prevalence fluctuated significantly between the months (Table 2), using January as the reference month, malaria infection risk showed a clear seasonal pattern. The highest odds of infection were observed during the post-monsoon period, with September showing the strongest association (OR = 4.04, 95% CI: 3.05–5.34, *p* < 0.001), followed by November (OR = 3.36, 95% CI: 2.28–4.94, *p* < 0.001) and October (OR = 3.13, 95% CI: 2.29–4.27, *p* < 0.001). Elevated infection risk was also observed in August (OR = 1.96, 95% CI: 1.49–2.58, *p* < 0.001), December (OR = 1.73, 95% CI: 1.16–2.58, *p* = 0.007), July (OR = 1.61, 95% CI: 1.23–2.09, *p* < 0.001), and June (OR = 1.55, 95% CI: 1.17–2.05, *p* = 0.002). In contrast, malaria infection odds were significantly lower in March (OR = 0.34, 95% CI: 0.22–0.54, *p* < 0.001) and February (OR = 0.61, 95% CI: 0.42–0.88, *p* = 0.008). No significant association was observed for April and May, suggesting that malaria transmission increased markedly during the warmer and post-monsoon months.

Year-by-year analysis showed that the prevalence of malaria was on the decline, with 39.5% in 2024 and 32.8% in 2025. The probabilities of infection were significantly lower in 2025 than in the baseline year (OR = 0.74, 95% CI: 0.68–0.81, *p* < 0.001), indicating possible advancements in malaria prevention measures over time.

### 3.3. Clinical Aspects Linked to Malaria

The univariate logistic regression evaluation found that clinical factors play a significant role in reducing malaria infection (Table 3). The recorded antimalarial treatment patterns showed that chloroquine (78.03%), artemether–lumefantrine (78.06%), and primaquine (77.69%) were among the most frequently administered drugs. These percentages are not mutually exclusive because some patients received combination therapy, particularly *P. vivax* cases treated with blood-stage antimalarial drugs along with primaquine. Treatment outcomes were recorded across all treatment categories; however, these findings represent documented treatment patterns and clinical management practices based on documented patient records rather than measures of therapeutic efficacy, as parasite clearance time, fever clearance time, follow-up outcomes and other efficacy indicators were not assessed. Compared with blood group A+, blood groups A, AB−, AB+, B−, B+, and O− were not significantly associated with the malaria infection (all *p* > 0.05). However, blood group O+ was associated with significantly higher odds of malaria infection (OR = 4.30, 95% CI: 1.10–16.86, *p* = 0.036).

None of the evaluated symptoms were significantly associated with malaria infection. Pregnancy was strongly associated with increased odds of the outcome, with pregnant individuals exhibiting nearly fourfold-higher odds compared with non-pregnant participants (OR = 3.98, 95% CI: 2.48–6.39, *p* < 0.001).

### 3.4. Multivariate Logistic Regression Analysis

Multivariate logistic regression analysis is presented in Table 4. In the adjusted analysis, gender and age remained significant predictors of malaria infection. Females exhibited significantly lower odds of malaria compared with males (AOR = 0.85; 95% CI: 0.78–0.94), indicating a modest but independent protective effect. Individuals aged 21–40 years (AOR = 0.64; 95% CI: 0.58–0.71), 41–60 years (AOR = 0.58; 95% CI: 0.47–0.71), and >61 years (AOR = 0.53; 95% CI: 0.27–0.90) had significantly lower odds compared with the 1–20-year reference group, indicating higher infection risk among younger individuals.

Pregnancy status was associated with malaria infection. Pregnant women had substantially higher odds of malaria compared with non-pregnant women (AOR = 4.96; 95% CI: 2.95–8.34; *p* = 0.001).

With blood group A+ as the reference, a clear differential association between ABO blood groups and malaria infection was observed. Blood group B demonstrated significantly reduced odds of malaria among both B+ (AOR = 0.77; 95% CI: 0.64–0.93) and B− individuals (AOR = 0.78; 95% CI: 0.67–0.92). A modest but statistically significant reduction in risk was also noted for the AB− blood group (AOR = 0.86; 95% CI: 0.75–0.99). Although lower odds were observed for A− and AB+, these associations were not statistically significant. In contrast, O+ and O− blood groups showed higher but non-significant odds of malaria infection, with wide confidence intervals indicating limited precision. Overall, these findings suggest blood group-specific susceptibility to malaria, with blood group B conferring a relative protective advantage.

In the adjusted model, calendar month remained a robust independent determinant of malaria infection, confirming pronounced seasonality in transmission. Using January as the reference, the odds of malaria were significantly elevated in February (AOR = 2.25; 95% CI: 1.69–3.01) and March (AOR = 2.31; 95% CI: 1.52–3.51), indicating an early seasonal increase. No statistically significant associations were observed during April (AOR = 0.76; 95% CI: 0.52–1.11) or May (AOR = 1.20; 95% CI: 0.83–1.74). A renewed rise in malaria risk was evident in June (AOR = 1.81; 95% CI: 1.37–2.38) and July (AOR = 1.83; 95% CI: 1.37–2.45), followed by a significant reduction in August (AOR = 0.39; 95% CI: 0.25–0.62). The strongest associations were observed in the post-monsoon period, with markedly increased odds in October (AOR = 3.78; 95% CI: 2.53–5.66), November (AOR = 3.78; 95% CI: 2.73–5.24), and December (AOR = 4.97; 95% CI: 3.71–6.66). Collectively, these findings demonstrate a heightened risk during late winter and post-monsoon months, likely reflecting the combined effects of rainfall, temperature, and vector dynamics.

In the multivariable model, preventive and environmental factors showed differential associations with malaria infection. The presence of a household water reservoir was not independently associated with malaria risk (AOR = 1.04; 95% CI: 0.93–1.15), suggesting limited contribution after adjustment for confounding variables. However, after adjustment for potential confounding factors, absence of mosquito net use was strongly associated with increased odds of malaria (AOR = 6.17; 95% CI: 4.96–7.67), underscoring the substantial protective effect of bed nets in this setting. Annual insecticidal spraying remained significantly associated with lower malaria odds (AOR = 0.08; 95% CI: 0.06–0.10).

### 3.5. Species-Specific Antimalarial Treatment Pattern

**Figure 3:**
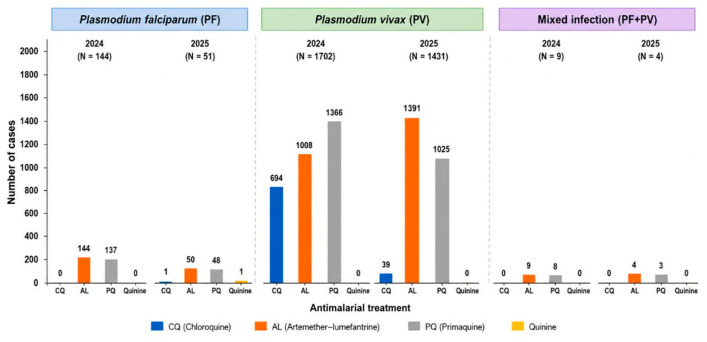
Species-specific antimalarial treatment patterns in RDT-confirmed malaria cases in northwestern Pakistan, 2024–2025.

Epidemiological analysis of rapid diagnostic test-confirmed malaria cases in northwestern Pakistan demonstrated clear species-specific infection patterns and associated treatment practices during 2024–2025 (Figure 3). *P. vivax* was the dominant species, with 1702 cases in 2024 and 1431 cases in 2025, whereas *P. falciparum* occurred less frequently (144 and 51 cases, respectively), and mixed infections were rare (9 and 4 cases). Treatment practices showed strong adherence to artemisinin-based combination therapy, with Artemether–lumefantrine used in nearly all *P. falciparum* and mixed infections and increasingly adopted for *P. vivax*. Notably, *P. vivax* treatment shifted substantially from a mixed regimen of artemether–lumefantrine (1008 cases) and Chloroquine (694 cases) in 2024 to a clear predominance of artemether–lumefantrine (1391 cases) with minimal chloroquine use (39 cases) in 2025. Primaquine was widely administered as adjunct therapy, particularly for *P. vivax* infections, reflecting its role in radical cure by targeting liver-stage hypnozoites and reducing the risk of relapse. These findings represent antimalarial treatment patterns and recorded clinical management practices among RDT-confirmed cases. Treatment efficacy was not assessed in this study because parameters such as parasite clearance time, fever clearance time and recurrence monitoring were not evaluated.

## 4. Discussion

In impoverished nations like Pakistan, malaria is still seen as a serious health concern [1,2], and high malaria infection rate was recorded in the present study. Compared to other Pakistani provinces, the Punjab region reports fewer than 10% of cases with roughly 52% of the population [22]. However, more than 30% of malaria cases occur in Baluchistan province, which has a population of 5%, while Sindh province, which has a population of 25%, accounts for almost 30% of all malaria cases [23,24]. Pakistan’s National Malaria Control Program focuses on reducing malaria burden through early diagnosis, prompt effective treatment, strengthened surveillance, integrated vector management, and targeted interventions in high-risk endemic areas. The predominance of *P. vivax* and observed changes in antimalarial treatment practices in the present study emphasize the importance of continued species-specific diagnosis, appropriate case management, and robust surveillance systems to support national malaria control and elimination objectives.

The results found *P. vivax* was to be higher (34%) in the current study than *P. falciparum* (2.1%), which is consistent with prior studies that found *P. vivax* to be the dominant species [25]. Our findings on *P. vivax*, which is more common in this study, are similar to those of other investigations carried out in other parts of Pakistan [13,26,27] but contrasting with another study where *P. falciparum* was more prevalent than *P. vivax* [3,12]. These differences may be due to different collection times such as *P. falciparum* frequency peaks between August and December while *P. vivax* peaks between April and September [28,29]. The increase or persistence of *Plasmodium* incidences in certain regions may have been influenced by cross-border migration [12].

Males (38%) had higher rates of *Plasmodium* infection in our study, which is consistent with prior research done in Khyber Pakhtunkhwa [13,30]. Males may be more exposed than females [31,32]. Patients under the age of 20 had 40% positive malaria cases, according to the current study, compared to those above the age of 20. However, healthcare facility-based recruitment may also influence age distribution, as caregivers may be more likely to seek testing for symptomatic children. According to our results, the frequency of malaria differs significantly among age groups. This is similar to other hyperendemic areas in sub-Saharan Africa where children below the age of five years are the disproportionate burden of the disease [33,34]. However, previous reports in South Asia and Latin America reported low to moderate transmission, where malaria is more uniformly affecting all age groups because of limited cumulative exposure to acquire strong clinical immunity [35].

Pregnancy was significantly associated with malaria infection in the present study, with pregnant participants showing higher odds of malaria positivity among individuals seeking healthcare. This finding is biologically plausible, as pregnancy is associated with immunological changes, reduced cell-mediated immunity, and altered susceptibility to malaria infection, particularly due to the sequestration of infected erythrocytes in the placenta. Similar increased vulnerability of pregnant women to malaria has been documented in malaria-endemic regions [5,36,37]. However, as our study was based on healthcare-seeking participants, this association may also be influenced by differences in testing practices, healthcare utilization, and preventive intervention coverage.

The current investigation indicated that there is a considerable association between malaria severity and ABO blood group in terms of distribution. In our study, blood group B showed a protective association against malaria infection, while O+ and O− groups showed higher but non-significant odds of infection. However, previous studies have demonstrated that blood group O may protect against severe malaria manifestations by reducing rosetting and cytoadherence of parasitized erythrocytes, which are important mechanisms in malaria pathogenesis [38,39,40]. Therefore, the higher non-significant odds of malaria infection observed among O+ and O− individuals in our study should be interpreted as an association with infection occurrence rather than severity. In A and B blood group antigens enhance rosette formation through PfEMP1 binding, whereas blood group O forms fewer and weaker rosettes due to the absence of these antigens [38]. AB blood group individuals express both A and B antigens, but its role in malaria susceptibility and severity remain unclear [40]. Further investigations incorporating clinical severity indicators are required to better understand the protective role of ABO blood groups against severe malaria outcomes.

Subdivisions were significantly associated with malaria infection based on unadjusted analysis, with the highest malarial cases in Kulachi and D.I. Khan and relatively fewer cases in Parova. However, Daraban and Paharpur showed higher proportions of positive cases relative to the number tested. The spatial distribution of malaria has been well documented and reflects local variations in the environment, mosquito breeding habitats, density of populations and coverage of the vector control programs [41]. Similarly, Afsheen [13] found tehsil-level malaria hotspots in Khyber Pakhtunkhwa linked with ecological and programmatic factors. A reduced level of burden in Parova may reflect lower risk of transmission or diagnostic underreporting associated with limited access to health facilities [18,42].

The malarial infection was significantly associated with location based on unadjusted analysis; rural areas carry a disproportionate amount of positive malaria cases compared to urban and peri-urban settings. This pattern is well documented in studies, as rural populations are constantly more exposed to vectors of mosquitoes due to poor housing, lack of preventive resources and proximity to breeding grounds [4,43]. Rural agricultural communities in Pakistan are also deemed areas with elevated risk for malaria [9,44]. The urban-rural divide might also be a sign of disparities in the distribution of preventive interventions such as insecticide-treated nets and residual spraying in the home [3,5].

The present result showed post-monsoon seasonality, consistent with seasonal malaria patterns in South Asia [45]. The same peaks of post-monsoon transmission have been observed in Pakistan, India and Nepal where monsoonal rainfall pushes predictable yearly epidemics as the conditions producing ideal breeding environments of *Anopheles* [45,46,47]. Pakistan’s monsoon-influenced landscape, during September–December and April–May, temperatures of 20–30 °C, combined with rainfall-generated breeding sites and high humidity, create favorable conditions for *Anopheles* development and malaria transmission [12]. Entomological studies have documented Pakistan’s malaria vector ecology, while climate variability and recurrent floods have further increased mosquito breeding habitats and transmission risks [48,49]. These seasonal dynamics have direct programming implications. Seasonally targeted measures, like indoor residual spraying, larviciding prior to peak transmission, and early monsoon delivery of ITNs, are recommended by WHO [1,3,5] and malaria control organizations in the area.

A significantly higher malarial positivity in 2024 than in 2025 was recorded; this may be partly related to increased transmission following the 2022–2023 floods in Pakistan, which created favorable conditions for malaria vectors. The decline observed in 2025 may reflect a reduction in post-flood transmission and ongoing malaria control efforts. However, this temporal decline may be due to improved treatment coverage, strengthened case management or enhanced vector control activities carried out during the research time frame. This could also be explained by changes in healthcare seeking behavior. Another possible contributor to the difference observed might have been inter-annual climatic variability impacting mosquito population dynamics [10,44]. Recognizing the influence of climate change on malaria resurgence is critical for developing adaptive control strategies and strengthening policy frameworks to achieve the ambitious target of a “Malaria-Free Pakistan by 2035” [50].

Our findings showed that primaquine and artemether + lumefantrine were the primary treatments for malaria infections in the health centers across the area. In total, 114 health centers 77.6% of malaria cases were treated with primaquine and 21.9% with chloroquine. Quinine was used to treat only one case, and artemether + lumefantrine was used to treat 78% cases. These percentages are not cumulative because some patients received combination therapy, particularly primaquine alongside blood-stage antimalarial treatment for *P. vivax* infections. In our study, artemether–lumefantrine was also used for the treatment of *P. vivax* cases. This reflects the use of artemether–lumefantrine in settings with suspected chloroquine resistance or according to local treatment practices. Although previous studies from Pakistan have reported treatment success rates and therapeutic responses for different antimalarial regimens, the present study evaluated treatment patterns rather than therapeutic efficacy. Therefore, these findings should not be interpreted as measures of drug effectiveness, as parameters such as parasite clearance time, fever clearance time, treatment failure, and molecular markers of resistance were not assessed in the current study. Previous malaria records from Punjab, Federally Administered Tribal Areas, Balochistan, and Sindh have reported variations in malaria distribution and species patterns. These regions are geographically distinct areas of Pakistan and are located outside the present study area of Khyber Pakhtunkhwa. According to reports from 2014, Punjab had a 58% recovery rate with chloroquine treatment, while Sindh and Balochistan had only a 17% cure rate [51]. In Balochistan, amodiaquine and sulfadoxine-pyrimethamine led to respective treatment success rates of 44% and 47%. In the Federally Administered Tribal Areas, 97% of patients received therapy with artesunate with sulfadoxine and pyrimethamine in 2014. Similarly, a 100% treatment success rate was achieved in 2016 in Sindh, Balochistan, and the Federally Administered Tribal Areas due to the tested effectiveness of artesunate + sulfadoxine-pyrimethamine [51]. Similarly, in the Federally Administered Tribal Areas, Sindh, Balochistan, and Khyber Pakhtunkhwa, the treatment rate with lumefantrine and artemether was 100% in 2017 [51]. In developing nations like Pakistan, where misdiagnosis of mixed species malaria infections can lead to inappropriate or insufficient treatment, choloroquine is used to treat *P. vivax* infections but is known to be inefficient against *P. falciparum* infections, better diagnosis and appropriate treatment are crucial for controlling malaria [52,53]. Artemisia species contain artemisinin and other bioactive compounds with promising antimalarial potential; however, their therapeutic application requires proper phytochemical characterization and standardization [54]. This is particularly important as the spread of antimalarial drug resistance and the absence of a highly effective vaccine continue to pose major challenges to malaria control, maintaining malaria as a critical global public health burden [55].

Occupational exposure based on unadjusted odds ratios plays a significant role in malaria risk in the study population. The literature is consistent that the most affected occupational groups are agricultural workers, livestock herders and construction labourers, because of their prolonged outdoor exposure during the peak mosquito biting hours of dawn and dusk [56]. This risk is biologically plausible because malaria vectors have demonstrated behavioral adaptations in response to control interventions, including shifts toward outdoor and early morning/evening biting, which increase human–vector contact and contribute to residual malaria transmission [57]. Therefore, occupation-specific preventive measures, including repellents, protective clothing, and targeted health education, are needed for high-risk groups.

There was a significant association between water reservoirs and malaria infection based on unadjusted analysis, emphasizing the role of water bodies as important mosquito breeding sites in the study area. This is in line with research from South Asia, where canals for irrigation, ponds and periodic flooded areas have been consistently linked to increased risk of malaria transmission [24,48]. Similarly, recent climatic and environmental events, particularly the 2022 floods in Pakistan, may have influenced malaria transmission dynamics. In northwestern Pakistan, flood-related stagnant water accumulation created favorable breeding conditions for *Anopheles* mosquitoes, while population displacement and disruption of healthcare services increased exposure risk and limited access to timely diagnosis and treatment. These factors may have contributed to increased malaria transmission in affected endemic communities [5,10,58]. Populations near water channels in D.I. Khan are said to be at higher risk of malaria due to the humidity conditions which are conducive for the development of mosquito larvae [13,42]. These findings endorse the implementation of conventional approaches to environmental management like improving drainage and removing stagnant water towards malaria vector control programmes [5].

Travel history has been recognized as an important contributor to malaria transmission based on unadjusted odds ratios in the present study. Movement of people between high-transmission areas and other regions, particularly in border and endemic areas, may increase exposure to malaria vectors and facilitate parasite introduction into new locations [3,5].

Annual insecticidal spraying showed a protective association with malaria infection, which is consistent with the role of indoor residual spraying in reducing *Anopheles* vector populations and transmission. This association likely reflects targeted implementation of spraying in high-transmission areas rather than a true protective effect of non-exposure. Overall, these findings highlight the dominant role of personal protective measures and programmatic targeting in shaping malaria risk. In the five endemic subdivisions of Dera Ismail khan, annual insecticidal spray was extremely low. Among the provinces, FATA showed the highest reported proportion (56%), followed by Balochistan (37.7%), Sindh (22.1%), and Khyber Pakhtunkhwa (15%) [59]. Another study found that only 30% of respondents in Pakistan reported using insecticide-treated nets (ITNs), with higher usage in Khyber Pakhtunkhwa (55%), highlighting gaps in the adoption of WHO-recommended vector control measures [26].

Mosquito net use showed a strong protective association in the multivariable model, but a non-significant association between mosquito net use and malaria risk in the crude analysis. This difference suggests the presence of confounding factors influencing the unadjusted relationship. Comprehensive interventions, including antimalarial medications, bed nets, and malaria health education, should be offered in the five endemic subdivisions.

The malarial sign and symptoms were not significantly associated with infection, consistent with the known clinical heterogeneity of malaria, from asymptomatic infection to severe febrile illness, thus symptom-based case detection is not reliable at the population level [15,31]. Common symptoms reported such as fever, chills, fatigue and headache overlap significantly with other endemic diseases such as typhoid and dengue [25]. Kouna [15] concluded that no single combination of symptoms is adequate for diagnosis without laboratory confirmation. Therefore, in malaria-endemic areas, suspected malaria cases should be confirmed using parasitological methods such as microscopy or rapid diagnostic tests before treatment decisions are made [3].

This study has some limitations, including the use of RDTs without microscopic or molecular confirmation, which limited parasite density assessment and detection of uncommon species. Healthcare facility-based recruitment may have introduced selection bias, and the two-year study period may limit evaluation of long-term epidemiological trends.

## 5. Conclusions

This study highlights substantial geographic and seasonal variation in malaria transmission in District Dera Ismail Khan, with *P. vivax* remaining the dominant species. Younger age, male sex, pregnancy, blood groups and inadequate preventive practices significantly influenced infection risk, emphasizing the need for targeted surveillance and strengthened vector control interventions before peak transmission periods. The observed transition from chloroquine to artemisinin-based combination therapy, particularly artemether–lumefantrine, reflects evolving species-specific treatment practices in the region. Malaria control efforts in South Asia are aligned with the WHO Global Technical Strategy for Malaria 2016–2030. Pakistan faces challenges including heterogeneous transmission, climate-related impacts, and population movement; therefore, strengthening surveillance, species-specific diagnosis, effective treatment, and vector control remains essential for progress toward elimination goals.

## Figures and Tables

**Table 1:** Sociodemographic and environmental factors of participants and their association with malaria infection.

Variable	Category	Total *n* (%)	Positive *n* (%)	Coefficient	OR (95% CI)	*p*-Value
Gender	Male (Ref)	4499 (48.8)	1723 (38)		1	
	Female	4712 (51.2)	1618 (34)	−0.1713	0.84 (0.77–0.91)	0.0001 ***
Age Group	1–20 (Ref)	5419 (58.8)	2177 (40)		1	
	21–40	3170 (34.4)	983 (31)	−0.4014	0.66 (0.61–0.73)	<0.001 ***
	41–60	555 (6)	161 (29)	−0.4967	0.60 (0.50–0.73)	<0.001 ***
	>60	67 (0.7)	20 (29.8)	−0.4562	0.63 (0.37–1.07)	0.0892 ^NS^
Locality	Outside (Ref)	179 (1.9)	42 (23)		1	
	Rural	7294 (79.2)	2789 (38)	0.7028	2.01 (1.42–2.86)	0.0001 ***
	Urban	1738 (18.9)	510 (29)	0.3036	1.35 (0.94–1.94)	0.0991 ^NS^
Tehsils	Adjacent areas (Ref)	179 (1.9)	42 (23)		1	
	D.I.K	2630 (28.6)	872 (33)	0.4812	1.61 (1.13–2.30)	0.0079 **
	Daraban	619 (6.7)	261 (42)	0.8663	2.37 (1.62–3.48)	<0.001 ***
	Kulachi	5382 (58.4)	2008 (37)	0.6634	1.94 (1.36–2.75)	0.0002 ***
	Paharpur	380 (4.1)	151 (39.7)	0.7659	2.15 (1.43–3.21)	0.0002 ***
	Parova	21 (0.2)	7 (33)	0.4892	1.63 (0.61–4.30)	0.3234 ^NS^
Occupation	Child (Ref)	2873 (31.2)	1177 (40.9)		1	
	Farmer	611 (6.6)	191 (31)	−0.4227	0.65 (0.54–0.78)	<0.001 ***
	Housewife	2332 (25.3)	708 (30)	−0.4649	0.62 (0.5–0.70)	<0.001 ***
	Job	52 (0.6)	21 (40)	−0.0242	0.97 (0.55–1.70)	0.9325 ^NS^
	Labour (Manual work)	1078 (11.7)	382 (35)	−0.2346	0.79 (0.68–0.91)	0.001 ***
	Other	295 (3)	103 (34.9)	−0.2575	0.77 (0.60–0.99)	0.0441 *
	Student	1970 (21.4)	759 (38)	−0.1019	0.90 (0.80–1.01)	0.0887 ^NS^
Travel History	No (Ref)	9144 (99.3)	3307 (36)		1	
	Yes	67 (0.7)	34 (50.7)	0.598	1.81 (1.12–2.94)	0.0148 **
Season	Dry (Ref)	8314 (90.3)	3001 (36)		1	
	Rainy	897 (9.7)	340 (37.9)	0.0776	1.08 (0.93–1.24)	0.2846 ^NS^
Bed Nets	No (Ref)	7297 (79.2)	2636 (36)		1	
	Yes	1914 (20.8)	705 (36.8)	0.0306	1.03 (0.92–1.14)	0.5656 ^NS^
Water reservoir	No (Ref)	2544 (27.6)	853 (33.5)		1	
	Yes	6667 (72.4)	2488 (37)	1.2044	3.33 (3.00–3.70)	<0.001 ***
Annual Spray	No (Ref)	7529 (81.7)	2978 (39.5)		1	
	Yes	1682 (18.3)	363 (21)	−0.8661	0.42 (0.37–0.47)	<0.001 ***

Ref: Reference category; ^NS^ not Significant; * *p* ≤ 0.05; ** *p* ≤ 0.01; *** *p* ≤ 0.001.

**Table 2:** Univariate logistic regression analysis of temporal factors associated with malaria infection among study participants.

Variable	Category	Total *n* (%)	Positive *n* (%)	Coefficient	OR (95% CI)	*p*-Value
Month	Jan (Ref)	319 (3.5)	81 (25)		1	
	Feb	395 (4.3)	68 (17)	−0.495	0.61 (0.42–0.88)	0.008 **
	Mar	305 (3.3)	32 (10)	−1.079	0.34 (0.22–0.54)	<0.001 ***
	Apr	340 (3.7)	76 (22)	−0.163	0.85 (0.59–1.21)	0.360 ^NS^
	May	965 (10.5)	250 (25.9)	0.03	1.03 (0.77–1.37)	0.855 ^NS^
	Jun	1131 (12.3)	391 (34.5)	0.438	1.55 (1.17–2.05)	0.002 **
	Jul	2599 (28.2)	919 (35)	0.476	1.61 (1.23–2.09)	<0.001 ***
	Aug	1294 (14)	518 (40)	0.673	1.96 (1.49–2.58)	<0.001 ***
	Sep	1061 (11.5)	614 (57.8)	1.396	4.04 (3.05–5.34)	<0.001 ***
	Oct	452 (4.9)	233 (51.5)	1.141	3.13 (2.29–4.27)	<0.001 ***
	Nov	180 (2)	96 (53)	1.212	3.36 (2.28–4.94)	<0.001 ***
	Dec	170 (1.8)	63 (37)	0.548	1.73 (1.16–2.58)	0.007 **
Year	2024 (Ref)	4688 (50.9)	1855 (39.5)		1	
	2025	4523 (49.1)	1486 (32.8)	−0.2913	0.74 (0.68–0.81)	<0.001 ***

Ref: Reference category; ^NS^ not Significant; ** *p* ≤ 0.01; *** *p* ≤ 0.001.

**Table 3:** Univariate logistic regression analysis of clinical characteristics linked to malaria infection among study participants.

Variable	Category	Total *n* (%)	Positive *n* (%)	Coefficient	OR (95% CI)	*p*-Value
Drugs						
CQ	No	2607 (78.03)	2607 (100)			
	Yes	734 (21.97)	734 (100)			
AL	No	733 (21.94)	733 (100)			
	Yes	2608 (78.06)	2608 (100)			
PQ	No	746 (22.32)	746 (100)			
	Yes	2595 (77.69)	2595 (100)			
Blood Group	A+ (Ref)	495 (5.4)	174 (35)		1	
	A−	1203 (13.1)	483 (40)	0.213	1.24 (1.00–1.54)	0.055 ^NS^
	AB−	1019 (11.1)	347 (34)	−0.049	0.95 (0.76–1.19)	0.673 ^NS^
	AB+	1862 (20.2)	642 (34)	−0.030	0.97 (0.79–1.20)	0.78 ^NS^
	B−	246 (2.7)	86 (34.9)	−0.008	0.99 (0.72–1.37)	0.959 ^NS^
	B+	4329 (47)	1580 (36)	0.059	1.06 (0.87–1.29)	0.555 ^NS^
	O−	47 (0.5)	22 (46.8)	0.485	1.62 (0.89–2.96)	0.115 ^NS^
	O+	10 (0.1)	7 (70)	1.46	4.30 (1.10–16.86)	0.036 *
Symptoms	Abdominal pain with Fever (Ref)	1369 (14.9)	499 (36)		1	
	Fever	4796 (52.1)	1740 (36)	−0.0073	0.99 (0.87–1.12)	0.9083 ^NS^
	Fever with Chills	1515 (16.4)	539 (35.5)	−0.0379	0.96 (0.82–1.12)	0.626 ^NS^
	Joint pain with Fever	1531 (16.6)	563 (36.7)	0.0139	1.01 (0.87–1.17)	0.8568 ^NS^
Pregnancy	No (Ref)	9130 (99.1)	3285 (35.9)		1	
	Yes	81 (0.9)	56 (69)	1.3827	3.98 (2.48–6.39)	<0.001 ***

Ref: Reference category; ^NS^ not significant; * *p* ≤ 0.05; *** *p* ≤ 0.001.

**Table 4:** Multivariate logistic regression analysis showing adjusted odds ratios (AORs) and 95% confidence intervals for factors associated with malaria infection.

Predictor Variable	Adjusted Odds Ratio (95% CI)	*p*-Value
**Gender**		
F vs. M	0.85 (0.78, 0.94)	0.001 **
**Age groups**		
21–40 vs. 1–20	0.64 (0.58, 0.71)	<0.001 ***
41–60 vs. 1–20	0.58 (0.47, 0.71)	<0.001 ***
>60 vs. 1–20	0.53 (0.27, 0.90)	0.02 *
**Pregnancy**		
Yes vs. No	4.96 (2.95, 8.34)	0.001 ***
**Blood Groups**		
A− vs. A+	0.81 (0.64, 1.02)	0.08 ^NS^
B+ vs. A+	0.77 (0.64, 0.93)	0.006 **
B− vs. A+	0.78 (0.67, 0.92)	0.003 **
AB+ vs. A+	0.79 (0.58, 1.08)	0.14 ^NS^
AB− vs. A+	0.86 (0.75, 0.99)	0.04 *
O+ vs. A+	1.39 (0.75, 2.58)	0.29 ^NS^
O− vs. A+	2.88 (0.69, 11.9)	0.14 ^NS^
**Months**		
February vs. January	2.25 (1.69, 3.01)	<0.001 ***
March vs. January	2.31 (1.52, 3.51)	<0.001 ***
April vs. January	0.76 (0.52, 1.11)	0.16 ^NS^
May vs. January	1.20 (0.83, 1.74)	0.31 ^NS^
June vs. January	1.81 (1.37, 2.38)	<0.001 ***
July vs. January	1.83 (1.37, 2.45)	<0.001 ***
August vs. January	0.39 (0.25, 0.62)	<0.001 ***
September vs. January	1.19 (0.87, 1.61)	0.25 ^NS^
October vs. January	3.78 (2.53, 5.66)	<0.001 ***
November vs. January	3.78 (2.73, 5.24)	<0.001 ***
December vs. January	4.97 (3.71, 6.66)	<0.001 ***
**Water reservoir**		
No vs. Yes	1.04 (0.93, 1.15)	0.45 ^NS^
**Mosquito Nets**		
No vs. Yes	6.17 (4.96, 7.67)	<0.001 ***
**Annual Insecticidal Spray**		
No vs. Yes	0.08 (0.06, 0.10)	<0.001 ***

^NS^ not Significant; * *p* ≤ 0.05; ** *p* ≤ 0.01; *** *p* ≤ 0.001.

## Data Availability

All data generated in this study are included in the published article.

## Supplementary Materials

The following supporting information can be downloaded at: https://www.mdpi.com/article/10.3390/tropicalmed11070194/s1, Supplementary File S1. Questionnaire.
