# Supplementary material for: Malaria Epidemiology and Plasmodium Species–Specific Antimalarial Treatment Patterns Among RDT–Confirmed Cases in Northwestern Pakistan
Source: Trop Med Infect Dis. 2026 Jul 10;11(7):194. doi: 10.3390/tropicalmed11070194 (PMC13417128; doi:10.3390/tropicalmed11070194)
Supplement: Supplementary file 1 [file tropicalmed-11-00194-s001.zip › tropicalmed-4392146-supplementary.pdf]

Supplementary file S1:

Quaid-i-Azam University Islamabad
Faculty of Biological Sciences
Department of Zoology

Questionnaire for Epidemiological and Molecular Study of Malaria in District Dera Ismail Khan, Khyber Pakhtunkhwa, Pakistan.

S.No.	Parameter	Question / Response Options
1	Gender	☐ Male ☐ Female ☐ (if Pregnant / Lactating) ☐ Other
2	Blood Group	________ (A+/A−/B+/B−/AB+/AB−/O+/O−)
3	Age (in years)	_____M / ________Y
4	Locality	☐ Urban ☐ Rural
5	Tehsil/UC	
6	Occupation	☐ Child ☐ Business ☐ Student ☐ Farmer ☐ Labour ☐ House-wife ☐ Job ☐ Shepherd ☐ Other
7	Malaria Infection History	☐ Once ☐ Repeated ☐ None
8	Antimalarial Drug Used	___________________ (e.g., Chloroquine, Artemether, Lumefantrine, Primaquine/ Quinine, ACTs etc.)
9	Use of Mosquito Nets at Night	☐ Yes ☐ No
10	Presence of stagnant water around the home?	☐ Yes ☐ No
11	How was malaria diagnosed?	☐ Rapid Diagnostic Test (RDT) ☐ Microscopy ☐ Clinical symptoms only
12	Season / Month	
13	Travel History	☐ Yes ☐ No
14	Symptoms	Malaria Symptoms Experienced ☐ Chills ☐ Fever ☐ Vomiting ☐ Muscle Ache ☐ Anemia ☐ Tiredness ☐ Diarrhea ☐ Recurrent fever every 24/48 hrs. ☐ Abdominal Pain ☐ Joint Pain
15	If Pregnancy, malarial infected	
16	Year	☐ 2024 ☐ 2025

Researcher Name: ______Aqsa Mansoor___________________________

Hospital/Center: __DHQ Hospital D.I.Khan_/D.I.Khan Clinic_______ __

Date: _____/_____/____
